# Prominent cerebral veins on susceptibility‐weighted angiography in acute meningoencephalitis

**DOI:** 10.1002/brb3.3255

**Published:** 2023-09-18

**Authors:** Yo Han Jung, Mina Park, Bio Joo, Sang Hyun Suh, Kyung‐Yul Lee, Sung Jun Ahn

**Affiliations:** ^1^ Department of Neurology, Gangnam Severance Hospital Yonsei University, College of Medicine Seoul South Korea; ^2^ Department of Radiology, Gangnam Severance Hospital Yonsei University, College of Medicine Seoul South Korea; ^3^ Severance Institute for Vascular and Metabolic Research Yonsei University College of Medicine Seoul South Korea

**Keywords:** acute meningoencephalitis, cerebral venous prominence, CSF glucose level, prominent cerebral veins, susceptibility‐weighted angiography

## Abstract

**Background and purpose:**

We have commonly observed prominent cerebral veins on susceptibility‐weighted angiography (SWAN) in acute meningoencephalitis. This study aimed to investigate the clinical significance of these findings.

**Methods:**

Cerebral veins on SWAN of 98 patients with acute meningoencephalitis diagnosed from February 2016 through October 2020 were classified into three groups according to the degree of venous prominence (mild, 23; moderate, 53; and prominent, 22). Clinical variables and laboratory findings were compared between these groups. The influence of variables on the prediction of prominent cerebral veins was measured by random forest (RF) and gradient boosting machine (GBM).

**Results:**

As cerebral veins became more prominent, cerebrospinal fluid (CSF) glucose level decreased (69.61 ± 29.05 vs. 59.72 ± 22.57 vs. 48.36 ± 20.29 mg/dL, *p* = .01) and CSF protein level increased (100.73 ± 82.98 vs. 104.73 ± 70.99 vs. 159.12 ± 118.15 mg/dL, *p* = .03). The etiology of meningoencephalitis, neurological symptoms, and increased intracranial pressure (ICP) signs differed between groups (*p* < .05). RF and GBM demonstrated that CSF protein level was the variable with the highest power to predict the prominent cerebral vein (mean decrease in node impurity: 4.19, relative influence: 50.66).

**Conclusion:**

The presence of prominent cerebral veins on SWAN in acute meningoencephalitis was significantly associated with a low CSF glucose level and a high CSF protein level, as well as ICP. Thus, the visual grade of the cerebral veins on SWAN may be utilized for the management of patients with acute meningoencephalitis.

## Graphical abstract

The presence of prominent cerebral veins on SWAN in acute meningoencephalitis was significantly associated with a low CSF glucose level and a high CSF protein level, as well as increased ICP. Thus, the visual grade of the cerebral veins on SWAN may be utilized for the management of patients with acute meningoencephalitis.

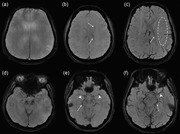

## 1 INTRODUCTION

Meningitis is an inflammation of the pia and arachnoid membranes covering the brain and spinal cord and requires prompt diagnosis and specific treatment because of its high mortality and morbidity (Jackson & Hayman, 2000; Kanamalla et al., 2000). Cerebrospinal fluid (CSF) analysis is the primary diagnostic method for suspected infectious meningitis; however, neuroimaging techniques such as magnetic resonance imaging (MRI) are crucial not only for diagnosis but also for monitoring therapeutic response (Lummel et al., 2016; Mohan et al., 2012). Particularly, postcontrast fluid‐attenuated inversion recovery (FLAIR) imaging has been known to be helpful in the diagnosis, mainly because of CSF suppression and high sensitivity to gadolinium leakage to the CSF space (Ahn et al., 2014; Kremer et al., 2006). Diffusion‐weighted imaging can also be used to confirm meningitis complications such as abscess and empyema earlier than other imaging modalities (Jan et al., 2003; Mohan et al., 2012).

Enhanced susceptibility imaging (ESI) techniques such as susceptibility‐weighted imaging (SWI), susceptibility‐weighted angiography (SWAN), and phase difference‐enhanced imaging (PADRE) have emerged as crucial techniques for detecting intracranial hemorrhage, venous thrombosis, and brain tissue at risk of infarction (Fujioka et al., 2013; Lou et al., 2014). Prominent cerebral veins have also been reported in acute stroke and interpreted as ischemic penumbra or poor collateralization of arterial supply (Luo et al., 2015; Verma et al., 2014). One case series has reported that a prominent cerebral vein may point to the area where migraine with aura originates (Karaarslan et al., 2011; Slavova et al., 2020). These phenomena can be explained by an uncoupling between oxygen supply and demand with a relative increase in the deoxyhemoglobin to oxyhemoglobin ratio (Mittal et al., 2009).

However, the clinical significance of ESI has not yet been assessed in patients with meningitis. We have serendipitously noted prominent cerebral veins as a common finding in patients with acute meningitis, prompting us to conduct this retrospective study to assess the relationship of meningitis with clinical and radiographic findings of prominent cerebral veins.

## 2 MATERIALS AND METHODS

### 2.1 Participants

This retrospective study was approved by our institutional review board, which waived the requirement for informed patient consent (3‐2021‐0423). We retrospectively searched electronic medical records and MR images between February 2016 and October 2020 and identified 132 patients with diagnoses of meningitis or encephalitis. We selected patients who had undergone a CSF study and MRI and were discharged with a diagnosis of meningitis or encephalitis. Ultimately, 98 patients were enrolled in the study. CSF studies and MRI were performed with an average gap of 1.73 days between MRI and CSF analysis (range, 0−18 days). Clinical data included age; sex; hypertension (HTN); diabetes mellitus (DM); smoking history; CSF analysis; neurological symptoms such as seizures, confusion, or aphasia; signs of increased intracranial pressure (ICP) such as a high CSF pressure, unconsciousness, abnormal pupillary dilatation, uncontrolled headache with nausea or vomiting, irregular breathing, and papilledema; duration of symptoms; duration of hospital admission; intensive care unit (ICU) care; and etiology of meningoencephalitis.

### 2.2 Image acquisition and analysis

MRI studies were performed using a GE 3T Discovery MR750 (GE Healthcare, Milwaukee, WI, USA). Our standardized protocol included T2‐weighted imaging, diffusion‐weighted imaging, FLAIR, susceptibility‐weighted angiography (SWAN), and subsequent contrast‐enhanced T1‐weighted imaging with FLAIR. The sequence parameters for SWAN were as follows: repetition time (TR) = 31 ms; echo time (TE) = 3 echoes centered around 23 ms; flip angle = 10°; slice thickness = 2 mm; intersection gap = 0 mm; field of view = 210 mm; matrix number = 320 × 224 and bandwidth = 62.50 kHz.

**FIGURE 1:**
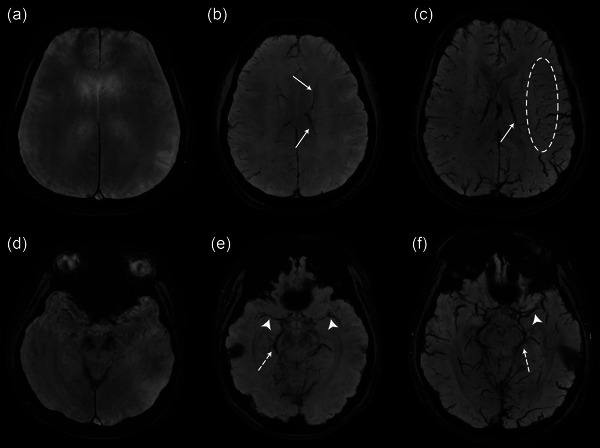
Grading system of prominent cerebral vein on minimum‐intensity‐projection image of SWAN. Grade 0 (mild, A, D)—superficial and deep parenchymal veins were rarely seen; Grade 1 (moderate, B, E)—subependymal veins (arrows) were clearly seen at the level of lateral ventricle. Deep middle cerebral vein (arrowhead) and basal vein of Rosenthal (dotted arrow) were clearly seen at the level of basal cistern. However, deep medullary veins were rarely seen; Grade 2 (prominent, C, F)—parenchymal veins, deep medullary veins (circle) and subependymal vein were clearly seen.

To enhance the visibility of veins and other sources of susceptibility, minimum intensity projection (mIP) of SWAN was acquired with a thickness of 8 mm in the axial plane. mIPs of SWAN sequences of all patients were assessed for the presence of prominent cerebral veins by two independent reviewers who were unaware of the clinical details. We used the three‐point score to evaluate the prominence of cerebral veins: Grade 0 (mild)—superficial and deep parenchymal veins were rarely seen; Grade 1 (moderate)—subependymal veins, deep middle cerebral vein, and basal vein of Rosenthal were clearly seen. However, deep medullary veins were rarely seen; Grade 2 (prominent)—parenchymal veins, deep medullary veins and subependymal vein were clearly seen (Figure 1).

### 2.3 Statistical analysis

Age, sex, HTN, DM, smoking history, CSF analysis (glucose level, protein level, and leukocyte count), blood tests (glucose level, protein level, and leukocyte count), CSF/serum glucose ratio, CSF/serum protein ratio, neurological symptoms (seizure, confusion, and aphasia), ICP signs, duration of symptoms, duration of hospital admission, ICU care, and etiology of meningoencephalitis were compared among the three groups with different cerebral vein grades on mIP of SWAN. A one‐way analysis of variance (ANOVA) with a post hoc Tukey's test was performed for continuous variables, while the chi‐square test or the Fisher's exact test was used for categorical variables.

For further analysis, the patients were dichotomized into two groups: patients with prominent cerebral veins and those without prominent cerebral veins (mild and moderate cerebral veins). To measure the influence of variables for the prediction of a prominent cerebral vein, the variable importance scores of each clinical variable were calculated using two popular ensemble methods: random forest (RF) and gradient boosting machine (GBM) (Breiman, 2001; Natekin & Knoll, 2013). These ensemble learning methods predict (using regression or classification) by combining the outputs from individual trees. RF refers to the process of creating and merging a collection of independent, parallel decision trees using different subsets of the training data, whereas GBM takes an iterative approach to combining several weak, sequential models into one strong model by focusing on the mistakes in the prior iterations. Statistical significance was set at *p* < .05. Interrater reliability was assessed using the intra‐class correlation coefficient (ICC) with a two‐way random model of absolute agreement. All data analyses were performed using R, version 3.5.3.

## 3 RESULTS

### 3.1 Patient characteristics and CSF analysis

**FIGURE 2:**
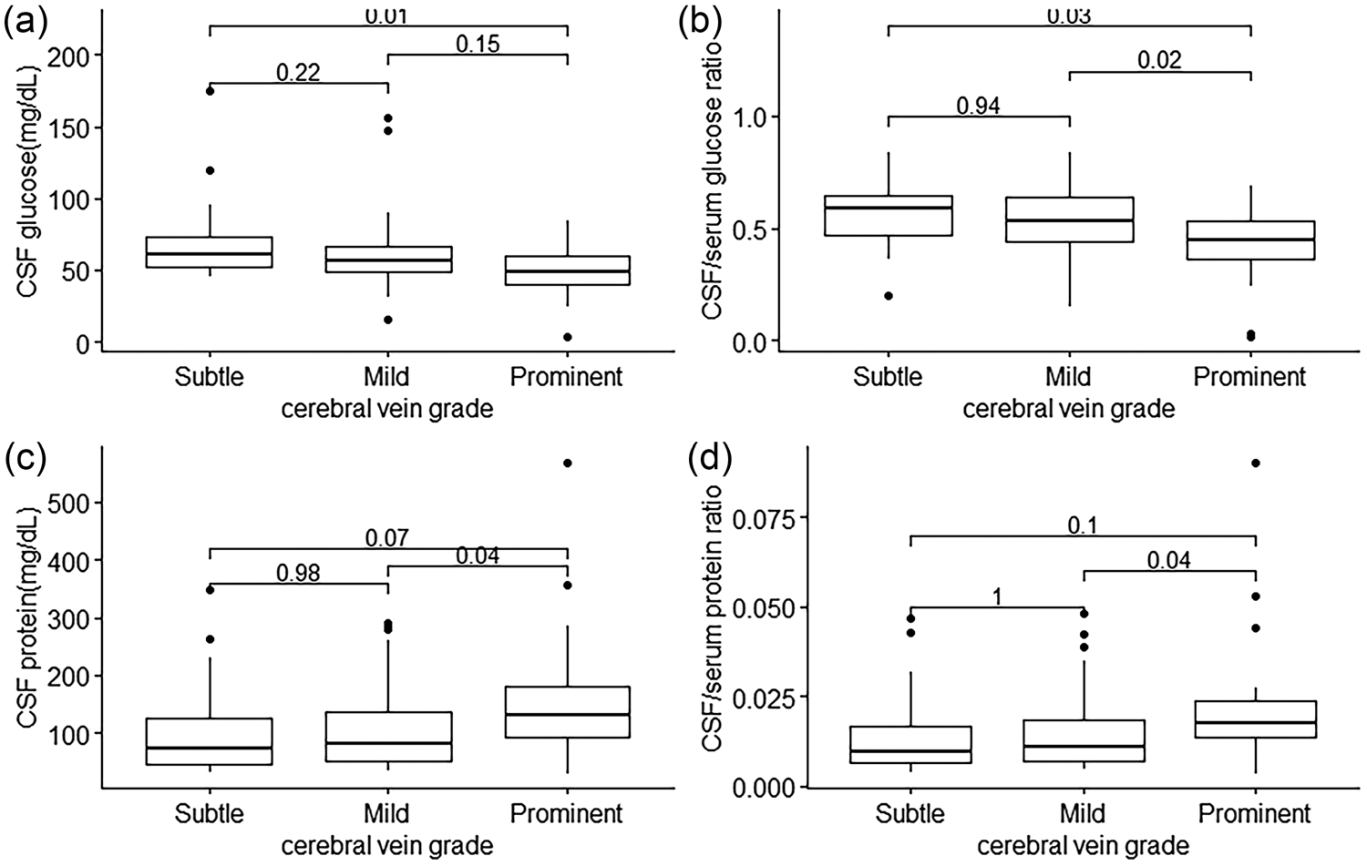
Boxplot of (A) CSF glucose level, (B) CSF/serum glucose ratio, (C) CSF protein, and (D) duration of admission days according to cerebral vein grade. Numbers indicate the results of post hoc *t*‐test.

Patient characteristics and CSF analyses are summarized in Table 1. There were statistically significant differences in CSF glucose level, CSF protein level, CSF/serum glucose ratio, CSF/serum protein ratio, etiology of meningoencephalitis, neurological symptoms, and ICP sign among patients with different cerebral vein grades (*p* < .05). As the cerebral veins became more prominent, the CSF glucose level and CSF/serum glucose ratio decreased. Post hoc analysis revealed that the CSF glucose level in patients with prominent cerebral veins was significantly lower than that in patients with mild cerebral veins (48.36 ± 20.29 mg/dL vs. 69.61 ± 29.05 mg/dL, *p* = .01; Figure 2A). The CSF/serum glucose ratio in patients with prominent cerebral veins was significantly lower than those in patients with mild (0.43 ± 0.18 vs. 0.56 ± 0.14, *p* = .03) and moderate cerebral veins (0.43 ± 0.18 vs. 0.54 ± 0.16, *p* = .02; Figure 2B).

**TABLE 1:** Baseline characteristics and CSF analysis of patients with meningitis or encephalitis according to prominence grades of cerebral vein.

Cerebral vein grade	Mild	Moderate	Prominent	*p* Value
	(*N* = 23)	(*N* = 53)	(*N* = 22)	
Sex				.52
F	11 (47.83%)	23 (43.40%)	7 (31.82%)	
M	12 (52.17%)	30 (56.60%)	15 (68.18%)	
Age	52.00 ± 20.30	40.58 ± 17.58	43.77 ± 20.87	.06
HTN				.62
No	17 (73.91%)	44 (83.02%)	18 (81.82%)	
Yes	6 (26.09%)	9 (16.98%)	4 (18.18%)	
DM				.11
No	18 (78.26%)	50 (94.34%)	19 (86.36%)	
Yes	5 (21.74%)	3 (5.66%)	3 (13.64%)	
Smoking				.75
Current	4 (17.39%)	9 (16.98%)	5 (22.73%)	
Ex	4 (17.39%)	7 (13.21%)	1 (4.55%)	
Non	15 (65.22%)	37 (69.81%)	16 (72.73%)	
CSF analysis				
Glucose (mg/dL)	69.61 ± 29.05	59.72 ± 22.57	48.36 ± 20.29	.01*
Protein (mg/dL)	100.73 ± 82.98	104.73 ± 70.99	159.12 ± 118.15	.03*
Leukocyte	196.13 ± 341.45	258.13 ± 308.40	575.41 ± 1153.42	.07
Blood test				
Glucose (mg/dL)	129.35 ± 48.55	113.60 ± 39.74	116.18 ± 24.05	.27
Protein (mg/dL)	6934.78 ± 756.55	7190.57 ± 530.70	7195.45 ± 600.38	.20
Leukocyte	11.37 ± 4.48	9.52 ± 6.96	9.93 ± 4.51	.46
CSF/serum glucose ratio	0.56 ± 0.14	0.54 ± 0.16	0.43 ± 0.18	.01*
CSF/serum protein ratio	0.01 ± 0.01	0.01 ± 0.01	0.02 ± 0.02	.04*
Etiology				.04*
Autoimmune	4 (17.39%)	2 (3.77%)	2 (9.09%)	
Bacteria	3 (13.04%)	1 (1.89%)	1 (4.55%)	
Fungus	1 (4.35%)	0 (0.0%)	2 (9.09%)	
Tuberculosis	0 (0.0%)	3 (5.66%)	1 (4.55%)	
Virus	5 (21.74%)	26 (49.06%)	7 (31.82%)	
Unknown	10 (43.48%)	21 (39.62%)	9 (40.91%)	
Neurologic symptom				.03*
No	0	33 (62.26%)	10 (45.45%)	
Yes	16 (69.57%)	20 (37.74%)	12 (54.55%)	
ICP				.04*
No	22 (95.65%)	42 (79.25%)	15 (68.18%)	
Yes	1 (4.35%)	11 (20.75%)	7 (31.82%)	
Duration of symptom (days)	6.96 ± 5.11	8.68 ± 11.96	8.73 ± 8.93	.77
Duration of admission (days)	25.78 ± 26.82	15.30 ± 14.28	26.18 ± 27.45	.05
ICU care				.10
No	18 (78.26%)	49 (92.45%)	21 (95.45%)	
Yes	5 (21.74%)	4 (7.55%)	1 (4.55%)	

ICU = intensive care unit; ICP = increased intracranial pressure.

*p value < .05.

As the cerebral veins became more prominent, the CSF protein level and CSF/serum protein ratio increased. Post hoc analysis revealed that CSF protein levels in patients with prominent cerebral veins were significantly higher than those in patients with moderate cerebral veins (159.12 ± 118.15 mg/dL vs. 104.73 ± 70.99 mg/dL, *p* = .04; Figure 2C). CSF/serum protein ratio in patients with prominent cerebral veins was also significantly higher than that in patients with moderate cerebral veins (0.02 ± 0.02 vs. 0.01 ± 0.01, *p* = .04; Figure 2D).

**FIGURE 3:**
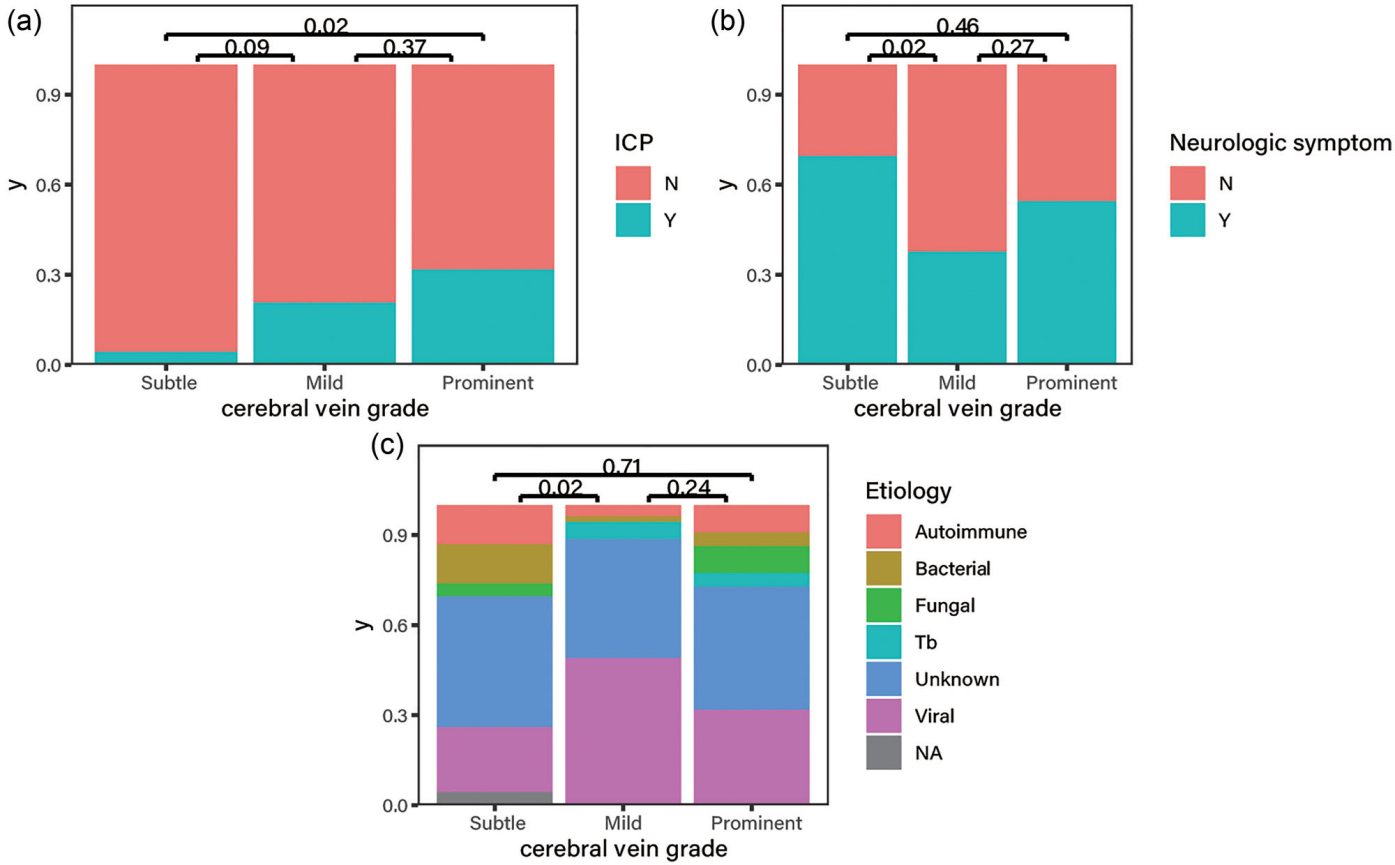
Stacked bar graphs of distribution of (A) ICP, (B) neurologic symptom, and (C) etiology according to cerebral vein grade. Numbers indicate the results of post hoc chi‐square test or Fisher's exact test.

ICP was observed more frequently for patients with more prominent cerebral veins (Figure 3A). Post hoc analysis revealed that patients with prominent cerebral veins were more likely to need a hyperosmotic agent than those with mild cerebral veins (31.82%, 7/22 vs. 4.35%, 1/23, *p* = .02). Patients with mild cerebral veins were more likely to have neurological symptoms than those with moderate cerebral veins (69.57%, 16/23 vs. 37.74%, 20/53, *p* = .02; Figure 3B). Autoimmune or bacterial etiology was noted more frequently in patients with mild cerebral veins than in those with moderate cerebral veins (30.4%, 7/23 vs. 5.66%, 3/53, *p* = .02; Figure 3C). No significant differences were observed in age, sex, HTN, DM, smoking history, blood test results, symptoms, duration of symptoms, duration of admission, or ICU care among the three groups. The interobserver agreement for the cerebral vein grade was excellent (ICC = 0.95).

In the ensemble methods, the variable with the highest predictive power for the prediction of the prominent cerebral vein was CSF protein level (mean decrease in node impurity: 4.19 in random forests, relative influence: 50.66 in gradient boosting), and the variable with the second highest prediction power was CSF glucose (mean decrease in node impurity: 3.07 in random forests, relative influence: 31.36 in gradient boosting). The importance scores of the other variables are summarized in Table 2.

**TABLE 2:** Variable importance associated with prominent cerebral vein using random forest and gradient boosting.

Random forest		Gradient boosting	
Mean decrease in node impurity		Relative influence	
CSF protein	4.19	CSF protein	50.66
CSF glucose	3.07	CSF glucose	31.36
Age	3.05	Age	9.54
Duration of admission days	2.35	ICP sign	5.65
ICP sign	0.68	Duration of admission days	2.77
Sex	0.37	Sex	0.00
Neurologic symptom	0.36	Neurologic symptom	0.00
Etiology	0.30	Etiology	0.00

## 4 DISCUSSION

In the present study, we found that cerebral venous prominence was associated with CSF glucose and protein levels. A prominent cerebral vein on SWAN indicated lower CSF glucose and higher CSF protein levels in patients with acute meningoencephalitis. Moreover, it was significantly associated with ICP. In contrast, when a cerebral vein is rarely seen, it may imply that patients are likely to have neurological symptoms.

Prominent cerebral veins on ESI have previously been described in ischemic regions of the brain in patients with stroke (Huang et al., 2012; Kesavadas et al., 2011; Xia et al., 2014). This phenomenon has been hypothesized to represent an uncoupling between oxygen and supply and demand within the hypoperfused tissue, with a relative increase in the deoxyhemoglobin to oxyhemoglobin ratio, which generates a local non‐uniform magnetic field, resulting in rapid dephasing of proton spins in ESI sequences (Kudo et al., 2016; Mittal et al., 2009). Thus, prominent cerebral veins are thought to imply either ischemic penumbra or poor collateralization of arterial supply (Luo et al., 2015; Sun et al., 2014). This sign has also been reported in patients with epilepsy and migraine (Karaarslan et al., 2011; Verma et al., 2016). However, cerebral venous prominence in acute meningoencephalitis has rarely been documented. In our study, it was commonly observed in patients with meningoencephalitis (22.4%; 22/98). Moreover, a prominent cerebral vein was significantly associated with low CSF glucose and high CSF protein levels. Meningitis causes vascular endothelial injury and breakdown of the blood–CSF barrier (Ashwal et al., 1992). Additionally, the fluctuation of cerebral blood flow has also been reported (Quagliarello & Scheld, 1992; Tunkel & Scheld, 1993). Meningeal inflammation with the release of a variety of factors, including oxygen free radicals, cerebral interstitial acidosis, and nitric oxide, is presumed to result in these hemodynamic changes. Our results suggest that a prominent cerebral vein sign may indirectly signal the breakdown of the blood–CSF barrier because an increase in CSF total protein level may result from greater permeability of the blood–CSF barrier (Sharief et al., 1992). As prominent cerebral veins are induced by reduced cerebral blood flow or vasoconstriction, a decrease in CSF glucose level can be explained by decreased glucose delivery to the CSF across the choroid plexus owing to reduced blood flow (Dzupova et al., 2009; Viola, 2010). In addition, our study demonstrated that prominent cerebral vein may indicate ICP, which may also increase cerebral venous outflow pressure, resulting in greater prominence of veins due to higher concentrations of intravenous deoxyhemoglobin (Higgins et al., 2002; Tong et al., 2008).

An additional noticeable finding of our study was that patients with a mild cerebral vein were more likely to display neurological symptoms. This contradictory phenomenon can be explained by followings. As inflammation progresses, the demand for oxygen can change, resulting in a low deoxyhemoglobin level. For example, diminished cerebral venous vasculature has been reported in patients with multiple sclerosis (MS) (Ge et al., 2009; Zivadinov et al., 2011) and is thought to be a result of decreased oxygen utilization in the chronic and widespread status of MS, leading to decreased levels of deoxyhemoglobin. Therefore, a diminished cerebral vein may reflect a worsened condition in patients with meningitis than an increased cerebral vein. We hypothesized that as meningitis progresses, visualization of cerebral venous structures may become normal and increase to decrease. However, to validate this hypothesis, more sophisticated stratification with a larger cohort is necessary in future studies.

The results of our study revealed another interesting finding: patients with mild cerebral veins had a higher proportion of autoimmune or bacterial meningoencephalitis compared to those with moderate cerebral veins. While this suggests a potential link between the prominence of cerebral veins and the type of meningoencephalitis, we also identified a noteworthy contradiction. The observed higher proportion of bacterial meningoencephalitis in mild cerebral veins seems to challenge the well‐established relationship between CSF glucose levels and bacterial meningitis, as bacterial meningitis is known to cause a significant decrease in CSF glucose levels (Tamune et al., 2014). The pathophysiological mechanisms and imaging features of autoimmune meningoencephalitis vary (Armangue et al., 2014; Budhram et al., 2019; Ding et al., 2021). Nevertheless, a considerable proportion of cases (39–43%) had unknown etiologies for meningitis in our study. Given the presence of unknown etiologies and the potential contradiction with CSF glucose levels, we must exercise prudence and avoid hasty conclusions when interpreting the significance of the prominence of cerebral veins in predicting the etiology of meningoencephalitis. Further research is warranted to explore additional factors that may contribute to a more comprehensive understanding of this relationship.

This study had limitations. First, there are different ESI techniques such as SWI, PADRE, and SWAN to visualize paramagnetic tissues. SWI and PADRE use phase information to increase the sensitivity with a different postprocessing technique (Haacke et al., 2009; Nandigam, 2013). SWAN technique modifies the T2*‐GRE by multiecho acquisition and calculates a weighted sum of the images obtained at different echo times. Shorter echo images lead to TOF effect with better anatomical images, whereas longer echoes are responsible for susceptibility effects (Ahn et al., 2014; Docampo et al., 2013). Thus, our results are based on the SWAN technique and may not be applicable in other ESI techniques. Second, although the interobserver agreement for visual assessment of cerebral vein was excellent, an automated quantification of the cerebral vein is necessary to increase the credibility of our results (Breiding et al., 2020). Third, the influence of oxygen administration on cerebral vein signals on SWAN was not evaluated. It is theoretically possible that the decrease in deoxyhemoglobin within cerebral veins due to oxygen inhalation could lead to a decrease in venous signal (Chang et al., 2014; Kesavadas et al., 2008). However, in our study a small portion of patients (10/98, 10%) were hospitalized in intensive care unit and might receive oxygen inhalation. Additionally, the assessment of patients' oxygen inhalation status was based indirectly on FLAIR images. Previous studies have reported that increased levels of oxygen inhalation can result in readily detectable cerebrospinal fluid (CSF) hyperintensity on FLAIR images (Anzai et al., 2004; Jeong et al., 2016). However, in our study, only one patient exhibited CSF hyperintensity on FLAIR images (Figure S1). Thus, it can be presumed that in our study, oxygen administration may not have significantly influenced the results. Fourth, there is a possibility that very few of patients have received hyperosmotic agents before taking MRI. The hyperosmotic agents can increase venous pressure by shifting water from brain into the blood vessels (Sabharwal et al., 2009). Thus, the significance of cerebral vein should be carefully interpreted in patients receiving a hyperosmotic agent before taking MRI.

In conclusion, our study demonstrated that a prominent cerebral vein shown on SWAN in acute meningoencephalitis patients was significantly associated with low CSF glucose levels, high CSF protein levels, and ICP. Moreover, contradictorily, a decreased cerebral vein may indicate worse patient outcomes, necessitating further research. Thus, the visual grade of the cerebral veins on SWAN may be utilized for the management of patients with acute meningoencephalitis.

## AUTHOR CONTRIBUTIONS

**Yo Han Jung**: Methodology, writing—original draft. **Mina Park**: Data curation, investigation. **Bio Joo**: Data curation, investigation. **Sang Hyun Suh**: Supervision, visualization. **Kyung‐Yul Lee**: Validation. **Sung Jun Ahn**: Writing—Reviewing and Editing.

## CONFLICT OF INTEREST STATEMENT

On behalf of all authors, the corresponding author states that there is no conflict of interest.

## FUNDING INFORMATION

The authors declare that no financial support was received for the research, authorship, and/or publication of this article.

### PEER REVIEW

The peer review history for this article is available at https://publons.com/publon/10.1002/brb3.3255.

## Supporting information

Figure S1. FLAIR image of 75‐year‐old female who had been hospitalized in intensive care unit. CSF hyperintensity (arrow) is noted on basal cistern (A) and prepontine cistern (B) on FLAIR.

## Data Availability

The data that support the findings of this study are available on request from the corresponding author. The data are not publicly available due to privacy or ethical restrictions.
