# Supplementary material for: Prominent cerebral veins on susceptibility‐weighted angiography in acute meningoencephalitis
Source: Brain Behav. 2023 Sep 18;13(11):e3255. doi: 10.1002/brb3.3255 (PMC10636387; doi:10.1002/brb3.3255)

Supplemental Figure 1. FLAIR image of 75-year-old female who had been hospitalized in intensive care unit. CSF hyperintensity (arrow) is noted on basal cistern (A) and prepontine cistern (B) on FLAIR.


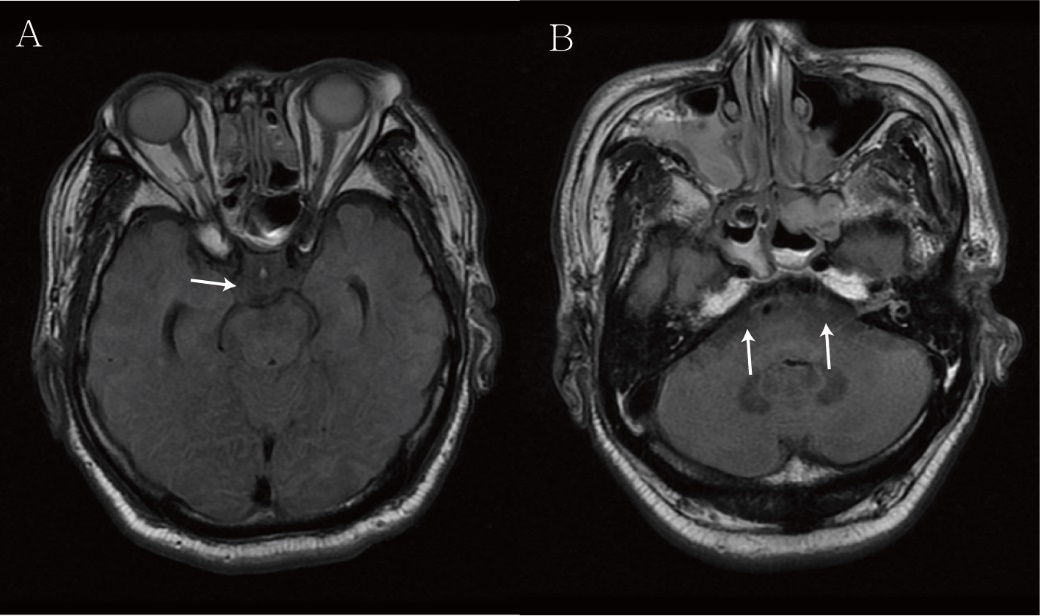

Supplement: Supplementary file 1 — Figure S1. FLAIR image of 75‐year‐old female who had been hospitalized in intensive care unit. CSF hyperintensity (arrow) is noted on basal cistern (A) and prepontine cistern (B) on FLAIR. [file BRB3-13-e3255-s001.docx]
